# Development of radiopharmaceuticals for targeted alpha therapy: Where do we stand?

**DOI:** 10.3389/fmed.2022.1020188

**Published:** 2022-12-22

**Authors:** Roger M. Pallares, Rebecca J. Abergel

**Affiliations:** ^1^Lawrence Berkeley National Laboratory, Chemical Sciences Division, Berkeley, CA, United States; ^2^Department of Nuclear Engineering, University of California, Berkeley, Berkeley, CA, United States

**Keywords:** targeted alpha therapy, radiopharmaceuticals, immunoconjugates, targeted radiotherapy, actinium-225

## Abstract

Targeted alpha therapy is an oncological treatment, where cytotoxic doses of alpha radiation are locally delivered to tumor cells, while the surrounding healthy tissue is minimally affected. This therapeutic strategy relies on radiopharmaceuticals made of medically relevant radionuclides chelated by ligands, and conjugated to targeting vectors, which promote the drug accumulation in tumor sites. This review discusses the state-of-the-art in the development of radiopharmaceuticals for targeted alpha therapy, breaking down their key structural components, such as radioisotope, targeting vector, and delivery formulation, and analyzing their pros and cons. Moreover, we discuss current drawbacks that are holding back targeted alpha therapy in the clinic, and identify ongoing strategies in field to overcome those issues, including radioisotope encapsulation in nanoformulations to prevent the release of the daughters. Lastly, we critically discuss potential opportunities the field holds, which may contribute to targeted alpha therapy becoming a gold standard treatment in oncology in the future.

## 1 Introduction

**FIGURE 1:**
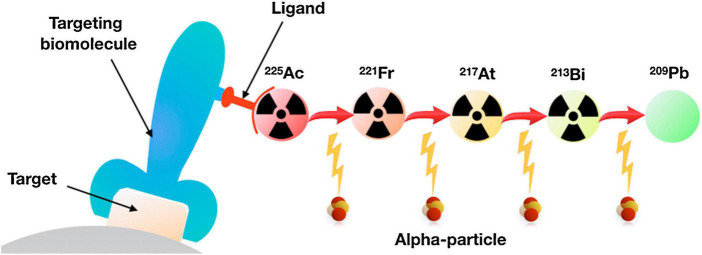
Schematic representation of targeted radiotherapy. In targeted alpha therapy, the targeting vector is conjugated with a ligand chelating an α-emitter, such as ^225^Ac, which emits four α-particles down to stable ^209^Pb. Adapted with permission from ref 2. Copyright 2018 Elsevier.

Therapeutic agents based on radionuclides hold great potential in oncology, as they allow to deliver highly cytotoxic doses of ionizing radiation to cancer cells, while minimizing damage to surrounding tissue (1, 2). Hence, targeted radiotherapy has been proposed to treat a wide range of cancers, including micrometastases, and tumors resistant to other treatments (3). Although targeted radiotherapy has been primarily explored in oncology, other potential uses include the treatment of viral and bacterial infections (4–7). In order to deliver the radioactive dose to the tumor site and spar healthy tissue, the radionuclides are complexed by chelating agents conjugated to targeting moieties, such as monoclonal antibodies (Figure 1) (1). To date, the U.S. Food and Drug administration (FDA) has approved two radioimmunoconjugate drugs to treat non-Hodgkin lymphoma: ^90^Y-ibritumomab tiuxetan and ^131^I-tositumomab, sold under the commercial names of Zevalin and Bexxar, respectively (8, 9), which rely on β-particle emissions. The latter was discontinued in 2014 due to manufacturing and commercial issues. Furthermore, the FDA and the European Medicines Agency (EMA) have also approved two targeting radiotherapeutics based on small molecules or peptides. The two radiopharmaceuticals are ^177^Lu-vipivotide tetraxetan (previously known as ^177^Lu-PSMA-617, and sold under the commercial name of Pluvicto) and ^177^Lu-oxodotreotide (sold under the commercial name of Lutathera) for the treatment of prostate-specific membrane antigen (PSMA)-positive metastatic castration-resistant prostate cancers and certain digestive tract cancers, respectively (10, 11).

The early success of β-emitters in cancer therapeutics has brought attention to α-emitting radionuclides, since they can potentially deliver greater and more confined cytotoxic dose. α-particles have higher linear energy transfer (50–230 keV/μm), which causes DNA break clusters, compared to β-particles (0.2 keV/μm), which yield individual and repairable DNA lesions (12). Studies with new-generation microbeam devices have further demonstrated that α-radiation cytotoxicity is also mediated by disruptions of other subcellular targets beyond the nucleus, including mitochondria, lysosomes, and cell membranes (13). In addition to the direct cytotoxicity of α-particles, α-emitters also cause biological effects through immunological and bystander effects (14, 15). Despite some of these mechanisms of action, such as bystander effects, depend on the generation of reactive oxygen species (16), high linear energy transfer radiation is less sensitive to oxygenation level and cell proliferation. Hence, the damage caused by α-emitters is more difficult to overcome than the one caused by β-emitters. Moreover, α-particles have shorter path lengths in biological tissues than β-particles do (50–100 and 1,000–10,000 μm, respectively), limiting the delivered dose to a narrower region (17). Hence, the dose confinement of α-particles may help to minimize cytotoxic damage outside the tumor region. Currently, only one α-emitting agent has been approved by the FDA, namely radium-223 dichloride, sold under the commercial name of Xofigo (formerly Alpharadin) (18). ^223^RaCl_2_ is used for the treatment of prostate cancer with metastatic bone disease, however, it does not rely on targeting agents to be delivered to the tumor site. Instead, radium acts as a calcium-mimetic cation that binds to hydroxyapatite deposition regions, including bone metastases in prostate cancer patients (19, 20). Although β-emitters, such as ^89^Sr and ^153^Sm, only provide pain palliation in bone-metastatic prostate cancer patients (21, 22), ^223^Ra yields both survival benefits as well as pain reduction when added to best standard of care (23, 24). Thus, ^223^Ra results exemplify the benefits of α-radiation compared to β-radiation in oncological settings.

Regarding targeted alpha therapy, early works focused on evaluating the therapeutic performance of immunoconjugates chelating single α-emitting isotopes (25). In recent years, however, radionuclides that emit multiple α-particles in the decay chain have received increasing interest, since they act as *in vivo* α-generators, enhancing the delivered dose (26). Hence, most ongoing clinical trials explore the use of radiopharmaceuticals radiolabeled with ^225^Ac or ^227^Th (Table 1). Although pre-clinical and clinical studies have highlighted the therapeutic benefits of these conjugates, there are still challenges that need to be solved, such as kinetics and stability of the complexes (27), and retention of the daughters (28, 29). In an α-decay, the recoiling daughter breaks the chemical bonds by which it is bound to the ligand. Free radioisotopes and metals, particularly f-block elements, have high binding affinities for biological receptors (30, 31), resulting in their endogenous chelation and subsequent deposition in tissues (32, 33). Internal contamination with radiometals (and their decay products) cause metal- and radiotoxicity, as multiple biological processes get disrupted (34–39). Therefore, minimizing daughter release is a fundamental step to extend the use of targeted alpha therapy beyond metastatic non-responding patients (28). While the design of new ligands is unlikely to solve the recoil issue (as the energies that the chelators need to withstand are too large), new nanoformulations, which encapsulate the α-emitting isotopes, have shown to enhance daughter retention (40, 41).

**TABLE 1:** Overview of ongoing targeted alpha therapy clinical trials.

Radiopharmaceutical	Ligand	Cancer type	Special notes	Clinical trial*
^211^At-BC8-B10	BC8-B10, antibody targeting CD45	Different types of acute leukemia or myelodysplastic syndrome		NCT03128034, phase I/II, recruiting (2017) NCT03670966, phase I/II, recruiting (2019) NCT04083183, phase I/II, recruiting (2020)
^225^Ac-Lintuzumab	Lintuzumab, antibody targeting CD33	Acute myeloid leukemia	In combination with other chemotherapeutic agents	NCT03441048, phase I, recruiting (2018) NCT03867682, phase I/II, recruiting (2020) NCT03932318, phase I/II, not yet recruiting (2023)
^212^Pb-DOTAMTATE	DOTAMTATE, somatostatin analog	Somatostatin positive neuroendocrine tumors		NCT03466216, phase I, recruiting (2018) NCT05153772, phase II, recruiting (2021)
BAY2315497 (^227^Th)	Antibody targeting PSMA	Metastatic castration resistant prostate cancer	In combination with darolutamide	NCT03724747, phase I, active but not recruiting (2018)
^225^Ac-FPI-1434	FPI-1175, antibody targeting insulin-like growth factor-1 receptor (IGF-1R)	Advanced solid tumors		NCT03746431, phase I/II, recruiting (2019)
BAY2701439 (^227^Th)	Antibody targeting HER2	Advanced cancers expressing the HER2 protein		NCT04147819, phase I, recruiting (2020)
JNJ-69086420 (^225^Ac)	H11B6, antibody targeting human kallikrein-2 (hk2)	Advanced and metastatic prostate cancer		NCT04644770, phase I, recruiting (2020)
^225^Ac-J591	J591, monoclonal antibody against PSMA	Hormone-sensitive metastatic prostate cancer	In combination with androgen deprivation therapy	NCT04946370, phase I/II, recruiting (2021) NCT05567770, phase 1, not yet recruiting (2022)
^225^Ac-PSMA-I&T	PSMA-I&T, small molecule targeting PSMA	Castration-resistant prostate cancer		NCT05219500, phase II, recruiting (2021)
^211^At-OKT10-B10	OKT10, antibody targeting CD38	Plasma cell myeloma in patients undergoing stem cell transplantation	In combination with different chemotherapeutic agents and/or total body irradiation	NCT04466475, phase I, recruiting (2022) NCT04579523, phase I, not recruiting yet (2022)
^225^Ac-DOTA-M5A	M5A, anti-carcinoembryonic antigen (CEA) antibody	CEA positive advanced and metastatic colorectal cancer		NCT05204147, phase I, recruiting (2022)
^212^Pb-DOTAM-GRPR1	Gastrin-releasing peptide receptors (GRPR) antagonist	Several GRPR1-expressing tumors		NCT05283330, phase I, not recruiting yet (2022)
^225^Ac-DOTA-daratumumab	Daratumumab, antibody targeting CD38	Refractory plasma cell myeloma		NCT05363111, phase I, recruiting (2022)
^225^Ac-FPI-1966	Vofatamab, antibody targeting fibroblast growth factor receptor 3 (FGFR3)	FGFR3-expressing advanced solid tumors		NCT05363605, phase I/II, recruiting (2022)
RYZ101 (^225^Ac)	Somatostatin analog peptide	Somatostatin receptor expressing gastroenteropancreatic neuroendocrine tumors		NCT05477576, phase I/II, recruiting (2022)
^225^Ac-MTI-201	MTI-201, peptide targeting melanocortin 1 receptor (MC1R)	Metastatic uveal melanoma		NCT05496686, phase I, recruiting (2022)
^212^Pb-Pentixather	Pentixather, CXC-chemokine receptor 4 (CXCR4)-directed peptide	Atypical lung carcinoid tumors		NCT05557708, early phase I, not recruiting yet (2022)
			

*The year in the clinical trial row refers to the date when the clinical study was (or is expected to be) initiated.

In this review, we analyze the current progress in the development of radiopharmaceuticals for targeted alpha therapy, from *in vitro* studies to clinical settings. We describe different oncological challenges this therapy tries to overcome, and then identify the design principles of radiopharmaceuticals that allow to overcome those issues, including targeting vectors, radionuclides, and delivery formulations. Finally, we discuss future research opportunities that the field may hold as well as challenges that need to be solved before targeted alpha therapy becomes a mainstream treatment in the clinic.

## 2 Radionuclides used in targeted alpha therapy

### 2.1 Radium-223

It is the first α-emitting isotope FDA-approved to treat cancer (18). ^223^Ra has a half-life of 11.4 days, and it can be obtained from ^227^Ac generators (42). It emits four α-particles and two β-particles as part of its decay chain down to stable ^207^Pb. The multiple α-emissions provide higher cytotoxic (and potentially therapeutic) effects, but they make the radiolabeling more challenging. One of its main drawbacks is the generation of ^219^Rn gas during its decay (43, 44).

### 2.2 Actinium-225

It is a radionuclide with a half-life of 10 days that produces six daughters during its decay chain down to stable ^209^Bi (12). Each ^225^Ac decay results on four α-emissions with energies between 5.8 and 8.4 MeV. ^225^Ac is advantageous over the FDA-approved ^223^Ra because it does not emit high-energy Ɣ-radiation. Furthermore, the half-life in the several days range allows for radionuclide central production and subsequent distribution, rather than on-site generation. Current ^225^Ac use in targeted alpha therapy is limited by the lack of chelating ligands capable of withstand the recoil energies (45).

### 2.3 Thorium-227

It is a radionuclide with a half-life of 18.7 days that decays to ^223^Ra through α-emission (46). Because the decay chain of ^227^Th contains five α-emissions, the radionuclide can act as *in vivo* α-generator during targeted alpha therapy, enhancing the dose delivered to the tumors (26). As in the case of ^225^Ac, applications of ^227^Th are limited by the capacities of the chelating agents to withstand the high recoil energies.

### 2.4 Bismuth-212 and -213

^212^Bi is a radioisotope with a half-life of 60.55 min that decays down to stable ^208^Pb through ^208^Tl (36%) and ^212^Po (64%) (12), with both decay routes emitting α- and β-particles. ^212^Bi is obtained through the decay of ^228^Th, but its short half-life complicates the radiolabeling process and sample preparation. Nevertheless, recent developments in ^224^Ra (half-life of 3.6 days) generators have partially overcome this issue, since they can produce both ^212^Pb and ^212^Bi with good yields (47). In addition to ^212^Bi short half-life, the radionuclide is also challenging to work with because one of its daughters (^208^Tl) emits high energy Ɣ-radiation (2.6 MeV).

Regarding ^213^Bi, it has a half-life of 45.6 min and can be produced in an ^225^Ac and ^213^Bi generator, which yields clinically useful radionuclide for 10 days (12). As in the previous radioisotope in this list, ^213^Bi short half-life also limits the preparation of therapeutic radioimmunoconjugates (48). Nevertheless, ^213^Bi decay chain includes a 440 keV Ɣ-emission that can be used to image tumor uptake and calculate dosimetry (49).

### 2.5 Lead-212

It is a radionuclide with a half-life of 10.6 h, which is produced during the ^228^Th decay, and commonly obtained from ^224^Ra generators (47). ^212^Pb is a β-emitter that serves as *in vivo* generator of the clinically relevant ^212^Bi, extending the use of the latter beyond its 60.55 min half-life (50). Simulations based on a Monte Carlo model showed that ^212^Pb and ^225^Ac have similar relative biological effectiveness when considering the entire decay chains (51). ^212^Pb, however, presents similar challenges that ^212^Bi does, such as a decay chain that contains a daughter (^208^Tl) that emits high energy Ɣ-radiation (2.6 MeV) (52).

### 2.6 Astatine-211

Astatine is the heaviest naturally occurring halogen, and one of its isotopes (^211^At) was proposed, more than fifty years ago, as substitute of iodine isotopes during specific inactivation of sensitized lymphocytes (53). ^211^At decay is branched, where the first path (42%) is by α-emission, resulting in the production of ^207^Bi, which is followed by electron capture to stable ^207^Pb (12). The second route (58%) is by electron capture, yielding ^211^Po, followed by emission of α-particles to stable ^207^Pb. The radiological properties of ^211^At are favorable for targeted alpha therapy, since the half-life of ^211^At is 7.2 h [long enough for most radiolabeling procedures to obtain the radioimmunoconjugates (12)], more than 99% of ^211^At radiation energy originates from α-emissions (54), and one of its daughters (^211^Pu) emits X-rays (77–92 keV), which can be used for imaging (55). While the other radioisotopes used for targeted alpha therapy are metals and their radiolabeling rely on metalation processes, At is a halogen. Hence, ^211^At radiolabeling is based on reactions with stannyl derivatives, iodonium salts, or boronic derivatives, among others (56). Nevertheless, ^211^At use in targeted alpha therapy is limited by its low availability and supply (57).

## 3 Dosimetry

Although dosimetry was initially developed for protection against radiation (58), nowadays it is also used for optimization of radiotherapy. The biological effect of radiation depends on the absorbed dose, which is defined as the amount of energy absorbed per unit of tissue mass (59), the fractionation and the spread of the exposure, among others (60, 61). While radiobiology and dosimetry for external-beam radiotherapy are well-established, their direct extrapolation to targeted radiotherapy is problematic, as the characteristics of the latter are rather different (i.e., mixed and heterogeneous irradiation, long exposure times, and low absorbed dose rates) (62). Therefore, new radiobiological understanding and dosimetry tools specific to targeted radiotherapy are necessary. In this regard, despite patient-specific dosimetry is slowly being implemented in clinical settings (63), radiobiological knowledge is still lacking to meet certain clinical needs (64). To that end, recent coordinated efforts are calling to collectively promote and foster advances in radiobiology with the aim to improve targeted radiotherapy outcomes (64).

In the case of targeted alpha therapy, dosimetry calculations are more challenging than in other types of targeted radiotherapy, since the daughters need to be considered, and they may have different pharmacokinetic profiles and chemical properties (65). Hence, each decay down to the stable isotope needs to be assessed. Roeske et al. developed a model that predicts dosimetry of α-emitters, which takes into consideration multiple factors, including the decay site, the daughter half-lives and their potential biodistribution, the blood time, and the tumor uptake (65). Because most of radionuclides used in targeted alpha therapy emit Ɣ-radiation (or some characteristic x-ray or bremsstrahlung radiation), biodistribution and pharmacokinetic information can be obtained for dosimetry calculations through clinical imaging (25). The spatial resolution of those images, however, tends to be poor, due to the injected activity for targeted alpha therapy is lower than the one used for imaging, yielding subpar signal-to-noise ratios (25, 66).

## 4 Radiopharmaceutical development: From pre-clinical to clinical studies

**FIGURE 2:**
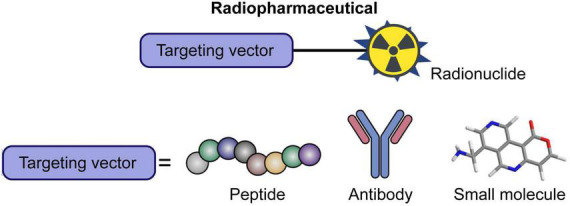
Schematic representation of radiopharmaceutical for targeted alpha therapy. The main targeting vectors are peptides, antibodies, and small molecules. Modified with permission from ref 45. Copyright 2020 Frontiers Media S.A.

In targeted alpha therapy, the radionuclides are delivered to cancer cells through a wide variety of formulations. Most radiopharmaceuticals are made of radiolabeled antibodies, peptides, or small targeting molecules (Figure 2) (26, 67). A recent strategy includes incorporating the radionuclides into liposomes or nanoconstructs as a mean to enhance tumor uptake and decrease daughters redistribution (41). The therapeutic performance of these nanoformulations, however, has only been studied in pre-clinical settings, and no clinical trials have been performed.

### 4.1 Small-molecule targeting

**FIGURE 3:**
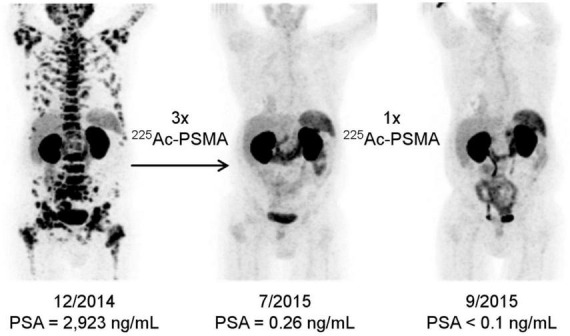
PET/CT scans of patient after receiving ^225^Ac-PSMA-617 treatments. Tumor spread before treatment **(left)**, restaging 2 months after third ^225^Ac-PSMA-617 cycle **(center)**, restaging 2 months after an additional consolidation cycle **(right)**. Adapted with permission from ref 53. Copyright 2016 Society of Nuclear Medicine and Molecular Imaging.

Over the last decade, increasing number of studies have focused on the use of small-molecule radiopharmaceuticals for targeting overexpressed antigens in cancer cells. For instance, PSMA is a type II membrane protein with enzymatic activity that is overexpressed on the cell membrane of aggressive prostate cancer and other solid tumors (68). Hence, PSMA is commonly targeted to deliver imaging and therapeutic agents to PSMA-expressing tumoral cells (69, 70). In the case of radiopharmaceuticals, PSMA-617 and PSMA I&T are two small-molecules that act as PSMA inhibitors, which are frequently radiolabeled with clinically relevant radionuclides (71, 72). For example, PSMA-617 labeled with ^177^Lu (β-emitter, half-life of 6.7 days) has shown promising therapeutic responses against metastatic castration-resistant prostate cancer in a phase II clinical trial (NCT03392428) (73), and is currently undergoing a phase III clinical trial (NCT03511664). The promising results with β-emitters promoted new research efforts toward developing PSMA-based alpha therapy. For instance, PSMA-617 radiolabeled with ^225^Ac showed therapeutic benefits in patients refractory to ^177^Lu-PSMA-617 (74, 75). The first clinical PSMA-based targeted alpha therapy study was published in 2016, where two patients with metastatic castration-resistant prostate cancer with challenging clinical situations received ^225^Ac-PSMA-617 (100 kBq/Kg) every 2 weeks (74). The first patient presented diffuse red marrow infiltration, yielding him unsuitable for ^177^Lu-PSMA-617 treatment, and the second one was resistant to the ^177^Lu radiopharmaceutical. Both patients experienced significant improvements after ^225^Ac-PSMA-617 treatments with prostate-specific antigen decreasing below measurable levels in serum, and complete response based on clinical imaging (Figure 3). It is worth highlighting that blood analysis and/or functional imaging (e.g., ^18^F-fluorodeoxyglucose-based positron emission tomography, PET) are important to characterize the therapeutic response after targeted radiotherapy, as the surface receptors may be downregulated after therapy, impairing molecular imaging. Furthermore, no hematologic toxicity was reported, and the only meaningful side effect was xerostomia. Based on the positive results, the same authors did a follow up study with 14 metastatic castration-resistant prostate cancer patients to optimize the treatment dose (76). For advanced-stage patients, a cycle of ^225^Ac-PSMA-617 (100 kBq/Kg) every 8 weeks showed the most optimal response when considering both therapeutic performance and toxicity. As the previous study, severe xerostomia was the dose-limiting side effect. Since then, the standardized treatment of ^225^Ac-PSMA-617 has been applied as last-line therapy to end-stage metastatic castration-resistant prostate cancer patients in other studies (75, 77). In one of those studies, 82% of chemotherapy-naïve patients showed above 90% serum prostate specific antigen decline, including 41% of patients having undetectable serum antigen levels, and remained in remission 12 months after the treatment (75). The first clinical data of ^225^Ac-PSMA-I&T have been published, showing comparable biochemical responses to ^225^Ac-PSMA-617 during the treatment of metastatic castration-resistant prostate cancer patients (78, 79). Recently, a pilot study with patients with metastatic prostate cancer, who received ^225^Ac-PSMA-617, reported two cases where patients developed ^225^Ac therapy-associated chronic kidney disease (80). Both patients had prior impaired renal function, which worsened after ^225^Ac therapy. This study highlighted the need to carefully assess and monitor kidney function on patients receiving ^225^Ac- PSMA-617, specially in cases with preexisting kidney impairment. An alternative to decrease therapy-associated toxicity, particularly xerostomia, is a tandem protocol, where both ^225^Ac-PSMA-617 and ^177^Lu-PSMA-617 are co-administrated. Tandem targeted therapy has shown similar initial response rates than ^225^Ac-PSMA-617 monotherapy (81), however, it still has stronger side effects compared to ^177^Lu-PSMA-617 monotherapy (82).

The PSMA-617 and PSMA I&T constructs have also been used to chelate other clinically relevant α-emitters, such ^213^Bi. For instance, Nonnekens et al. compared the double-strand DNA breaks induced by ^213^Bi-PSMA I&T and ^213^Bi-JVZ-008 (a PSMA nanobody) in mice bearing prostate cancer xenografts (83). ^213^Bi-PSMA I&T showed higher tumor uptake and double-strand DNA breaks than its nanobody counterpart. Regarding PSMA-617, there is only one clinical study of ^213^Bi-PSMA-617, where a patient with metastatic castration-resistant prostate cancer was treated with the radiopharmaceutical (84). The patient, who was progressive under conventional therapy, received two cycles of ^213^Bi-PSMA-617. Remarkable molecular imaging response was observed by PET after 11 months. Moreover, the patient biochemistry notably improved with prostate specific antigen levels decreasing from 237 μg/L down to 43 μg/L. Nevertheless, a follow up study, which estimated the dosimetry of ^213^Bi-PSMA-617, showed that although the radioconstruct can reach dose levels acceptable for clinical applications, it has higher perfusion-dependent off-target radiation than ^225^Ac-PSMA-617 (85).

**FIGURE 4:**
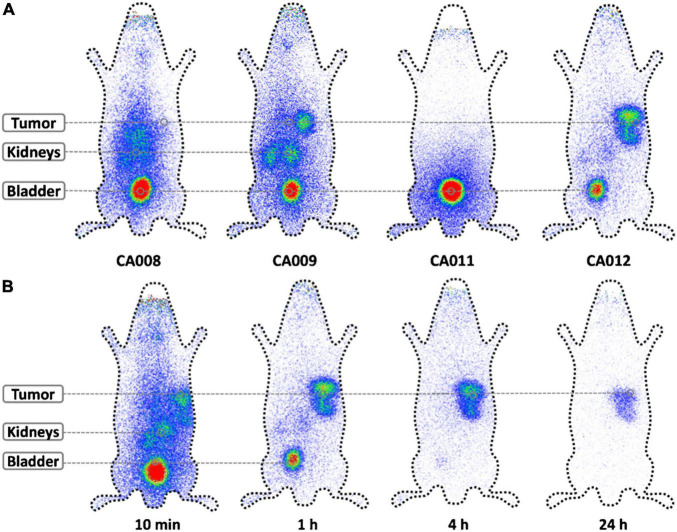
Theranostic pairs used for pharmacokinetics and dosimetry estimations. The Ɣ-emitting ^203^Pb was used as imaging surrogate of therapeutic ^213^Pb to estimate the pharmacokinetics and dosimetry of different PSMA-targeting radiopharmaceuticals. Planar scintigraphy scans of **(A)** different radioconjugates 1 h after injection, and **(B)** ^203^Pb-CA01 at different time points. Adapted with permission from ref 66. Copyright 2018 Springer Nature.

A novel strategy in the field of radiopharmaceuticals includes labeling the drugs with theranostic pairs, where one radionuclide provides therapeutic performance and the other one is used for imaging/diagnostic purposes (86, 87). As an example, PSMA-targeting ligands have been combined with ^212^Pb (parent of α-emitter ^212^Bi) and ^203^Pb (Ɣ-emitter with a half-life of 52 h), which can be used as agent for single photon emission computed tomography (SPECT) (88). Several new PSMA-targeting ligands have been developed for ^212^Pb and/or ^203^Pb, which provide rapid tumor uptake in mice bearing xenografts (89) and favorable antitumor responses (52). In clinical settings, the Ɣ-emitting ^203^Pb has been used as imaging surrogate to estimate the dosimetry of PSMA-targeting ^212^Pb drugs through planar scintigraphy scans (Figure 4) (90). ^211^At is another α-emitter that has shown significant tumor growth inhibition in xenograft models (91). This work has been recently expanded by developing new ^211^At therapeutic conjugates that display enhanced *in vivo* stability and tumor accumulation (92).

As mentioned earlier, the main dose-limiting side effect of radiopharmaceuticals using small molecules to target PSMA is severe xerostomia, which results from high salivary gland uptake (93). Recent awareness about xerostomia has shift radiopharmaceutical development toward other targeting vectors. For instance, Kelly et al. enhanced ^225^Ac ligand clearance by binding serum albumin to the PSMA-targeting molecules (94). Alternatively, anti-PSMA antibodies have also been used to target the antigen, maximizing selective tumor uptake, and preserving therapeutic performance *in vivo* (95).

### 4.2 Peptide targeting

The use of peptides in radiopharmaceuticals dates back to early 1990s, when somatostatin receptor targeting peptides were used in the clinical imaging of neuroendocrine and other somatostatin-positive tumors (26, 67). ^111^In-DTPA-DPhe1-octreotide was the first radiopharmaceutical to reach the market (96). Hence, the next natural step was to explore those same peptides for therapeutic applications. After initially studying the tumor suppression performance of ^111^In, which decays through electron capture, the field shifted toward other β-emitters, including ^90^Y and ^177^Lu (26). As a result of those efforts, ^177^Lu DOTATATE (an eight amino acid peptide covalently bonded to a DOTA chelator and labeled with ^177^Lu) was approved by the FDA for the treatment of somatostatin receptor-positive gastroenteropancreatic neuroendocrine tumors (97). The approval was based on a phase III clinical trial results (NCT01578239), which demonstrated ^177^Lu DOTATATE treatment yielded longer progression-free survival and higher response rate compared to standard of care. These results have encouraged new research toward developing similar formulations using α-emitters. For instance, DOTAMTATE (another somatostatin analog conjugated to a DOTA chelator unit) was labeled with ^212^Pb, and its therapeutic performance evaluated in animal models (98). After receiving three treatment cycles of 370 kBq ^212^Pb DOTAMTATE every 2 weeks, 79% of the mice were tumor free at the end of the 31-week study. Since then, a phase 1 clinical trial has been initiated to study ^212^Pb DOTAMTATE (commercially known as AlphaMedix) in adults suffering from neuroendocrine tumors (99). Preliminary results have shown favorable safety profiles, although full evaluations of safety and clinical performance are still in progress.

### 4.3 Antibody targeting

Antibodies have been the most commonly used targeting vector in targeted alpha therapy (27). Among the different classes of antibody to choose from, IgGs are the preferred ones because of their long circulation half-life (between 2 and 5 days depending on structure) and efficient elimination through liver and reticuloendothelial system (100). Moreover, antibody-based radiotherapy has benefited from current technology that allows to obtain IgGs with well-defined binding and selectivity against specific antigens (100). Similar to small molecule and peptide-based radiotherapy, early studies using antibodies as delivery vehicles focused on β-emitters, such as ^131^I (101, 102). More recently, the α-emitter ^211^At was explored as alternative to ^131^I during the ablation of bone marrow in preparation for transplantation. A synergistic treatment combining ^211^At-anti-CD45 immunoconjugates and bone marrow transplantation expanded survival in disseminated murine leukemia models (103). Minimal serological toxicity was observed after treatment, with recovery of white blood cell counts after 4 weeks. This study was followed by a second one that adjusted the reaction conditions and the quality control methods used to obtain the radioimmunoconjugate in laboratory settings to current good manufacturing practice production (as enforced by the FDA) (104). As a result of this progress, the ^211^At-radioimmunoconjugate has moved to a phase I/II clinical trial (NCT03128034) for the treatment of patients with relapsed or refractory high-risk acute leukemia before donor stem cell transplant.

Lintuzumab (also known as HuM195) is another monoclonal antibody used to target leukemia cells (105). Particularly, lintuzumab binds to a cell surface glycoprotein (CD33) found on the majority of myeloid leukemia cells, and myelomonocytic and erythroid progenitor cells (106). Hence, HuM195 has been one of the most explored antibodies for the development of radioimmunoconjugates against blood cancers. For example, an early pre-clinical work explored the dose-dependent cytotoxicity of ^213^Bi-HuM195 *in vitro* and the safety profile *in vivo* (107). The authors followed up with a study that characterized the pharmacokinetics and toxicity of ^213^Bi-HuM195 in mice (108), and another one that scaled up the production of the radioimmunoconjugate from pre-clinical to clinical quantities (109). All these pre-clinical studies provided enough data to move the development of the radiopharmaceutical to clinical settings, where the pharmacokinetics and dosimetry of ^213^Bi-HuM195 were evaluated in patients with leukemia (49, 110). The drug was also studied in a phase I dose-escalation trial that included 18 patients with advanced myeloid leukemias (111). The study showed reductions in bone marrow blast in 78% patients, although no complete remissions were reported. ^213^Bi-HuM195 dosage was further evaluated in another phase I/II trial, which determined the maximum tolerated dose and therapeutic effects after the patients had received chemotherapy (112). Because ^213^Bi is limited by its short half-life, subsequent studies with lintuzumab explored the use of other (longer-lived) radioisotopes, such as ^225^Ac (113, 114). Single doses of ^225^Ac-HuM195 in kBq range induced tumor regression and improved survival without toxicity in mice bearing tumor xenografts (113). Due to the release of ^225^Ac daughters during treatment had been associated with the development of radiation-induced nephritis (115), new strategies to protect renal function were developed. For instance, a low-dose spironolactone (a potassium-sparing diuretic) was administrated during treatment with ^225^Ac-HuM195, preventing the development of functional and histopathologic changes in the kidneys (116). Based on the pre-clinical data, two phase I clinical trials were undertaken, the first one was a dose escalation study to identify the safety, pharmacology, and biological activity of ^225^Ac-HuM195 (117), while the second one was a dose-escalation trial combining the radiopharmaceutical with chemotherapy (118). Since then, ^225^Ac-HuM195 has entered in a phase II clinical trial to establish the response rate in patients aged 60 years old and older with acute myeloid leukemia. Preliminary results showed a response rate of 69% in patients receiving 72 kBq/kg/dose (119). However, due to the high incidence (46%) of grade 4 thrombocytopenia, the dose was decreased to 55.5 kBq/kg for the rest of the clinical trial, which is currently ongoing. Beyond lintuzumab, daratumumab is another myeloma-targeting antibody (120), which has been recently explored for targeted alpha therapy. Nevertheless, although daratumumab radiolabeled with ^225^Ac has shown promising antitumoral effects both *in vitro* (121) and *in vivo* (122), the radiopharmaceutical has yet to move to clinical studies.

Beyond blood cancers, several radiopharmaceuticals have been developed to target solid tumors. For example, insulin-like growth factor receptor (IGF-1R) is an oncogenic protein over-expressed on the surface of a wide range of tumor cells (123). Because drugs targeting the IGF-1R had shown poor antitumoral effects in clinical settings (124), targeted alpha therapy was proposed as a therapeutic strategy against IGF-1R-expressing solid tumors. Hence, AVE1642 (a monoclonal antibody targeting IGF-1R) labeled with ^225^Ac was used to treat immunodeficient mice with colorectal, radioresistant lung, or prostate tumor xenografts (125). Single doses (between 1.85 and 14.8 kBq) showed high anti-tumor efficacy, as observed by the decrease of tumor volumes. Based on the positive *in vivo* data as well as previous clinical experience with the antibody itself, the first clinical trial (NCT03746431) was initiated to determine the pharmacokinetics and safety profile of the ^225^Ac radioimmunoconjugate (126).

Another antigen used in targeted alpha therapy against solid tumors is mesothelin, a membrane glycoprotein involved in cell proliferation that is overexpressed in ovarian, lung, pancreatic and triple-negative breast cancers, among others (127, 128). For example, a single dose administration (250 or 500 kBq/Kg) of a ^227^Th radioimmunoconjugate that targets mesothelin showed statistically significant antitumor effects in orthotopic bone xenograft models compared to control groups (129). Similar results were also reported when the ^227^Th radioimmunoconjugate was used to treat mice with other xenograft models, including breast, colorectal, lung, ovarian, and pancreatic tumors (130).

Trastuzumab is another IgG antibody used in the treatment of solid tumors, such as breast, ovarian, and gastric cancers (131). Trastuzumab targets human epidermal growth factor receptor 2 (HER2), a protein involved in cell proliferation that is overexpressed in multiple tumors [e.g., between 20 and 30% of breast cancers (132), and between 15 and 30% of ovarian cancers (133)]. Thus, trastuzumab (sold under commercial name of Herceptin) was approved by the FDA in 2008 to treat HER2-overexpressing metastatic breast cancers. However, the antibody as stand-alone treatment has limited efficacy, since the majority of tumors that initially respond to the treatment develop resistance within a year, becoming progressive again (134). Hence, radiotherapy using trastuzumab as targeting agent was proposed, since the radiopharmaceutical would likely require less antigens in each cancerous cell to be effective (compared to the antibody treatment), and resistance would be unlikely to occur. An early study explored the use of trastuzumab radiolabeled with ^213^Bi to treat colon and pancreatic xenograft models (135). Although the radiopharmaceutical had anti-tumor effects in both models, only mice bearing human colon carcinomas showed significant survival increases (from 20.5 days to 43 and 59 days after receiving doses of 18.5 and 27.75 MBq, respectively). A follow up study by the same authors compared the therapeutic performance of trastuzumab when radiolabeled with ^212^Bi, ^213^Bi, and ^212^Pb against peritoneal xenografts (136). ^212^Pb had better therapeutic index compared to the other two radionuclides, and required lower doses (between 370 and 1,480 kBq) to promote effective cytotoxic responses. The therapeutic benefits of ^212^Pb were also demonstrated in mice bearing human pancreatic carcinoma xenografts, which had been previously reported as unresponsive to ^213^Bi-trastuzumab (136). Moreover, the mouse survival could be further extended by combining the ^212^Pb-trastuzumab treatment with the administration of Gemcitabine (a chemotherapeutic agent) (137). All these pre-clinical data resulted on ^212^Pb-trastuzumab being explored on clinical settings. A phase I clinical study with three patients with HER2-expressing cancers that had been non-responsive to standard therapies received 7.4 MBq/m^2^ intraperitoneal injections of the radioimmunoconjugate to study pharmacokinetics and toxicity (138). Imaging demonstrated almost no radiopharmaceutical distribution outside the peritoneal cavity, while the administered dose was well tolerated by the patients with minimal toxicity signs. A follow up dose escalation and dosimetry study was performed by the same authors, showing minimal toxicity at more than a year after the patients received 7.4 MBq/m^2^, and almost no toxicity more than 4 months after the patients received 9.6, 12.6, 16.3, or 21.1 MBq/m^2^ (139). After a year, the authors reported that all dose levels were well tolerated with drug-related adverse effects being transient, mild, and not dose dependent (140). The mild side-effects included asymptomatic and abnormal laboratory values. However, no late renal, cardiac, or liver toxicity was observed up to a year post administration. Because all doses explored seemed safe, a higher dose (27 MBq/m^2^) was also investigated, and it was also well tolerated (140).

As the field of targeted alpha therapy started shifting from short-lived radionuclides to longer-lived ones, such as ^225^Ac and ^227^Th, so did the pre-clinical research using trastuzumab. An initial study demonstrated the therapeutic effect of trastuzumab radiolabeled with ^227^Th in breast and ovarian cancer cell lines (141). This study was followed by *in vivo* studies using different xenograft models, including ovarian, breast, and orthotopic bone cancers (46, 142–144). Normal tissue toxicity could be decreased by splitting the administrated dose (1000 kBq/kg) into several fractions (from a single injection to four injections every 2 or 4 weeks), while preserving the therapeutic effect (46). Furthermore, when the radiopharmaceutical was co-administrated with Olaparib (a chemotherapeutic drug), both treatments showed synergistic effects (145).

### 4.4 Nanoformulations

Liposomal and inorganic nanoconstructs are currently being investigated as delivery vehicles of radionuclides in pre-clinical settings (41, 146). Nanoformulations are advantageous over traditional delivery systems because they show enhanced cellular uptake (147, 148), high surface-to-volume ratios (149) that results in high loading capabilities, and ease of functionalization (150–152). Moreover, the unique optoelectronic properties of inorganic nanoparticles, which can be controlled through crystal engineering (153–156), can be exploited by other forms of therapy, such as photothermal therapy (157), magnetic hyperthermia (158), or smart drug-release (159), and imaging/diagnostics (160–163). Hence, nanoformulations that combine targeted alpha therapy with another type of therapy could potentially provide synergistic treatments.

One of the first nanoparticles explored for radionuclide delivery were zeolite nanoconstructs. A concern when using ^223^Ra in targeted alpha therapy is the recirculation of its daughters, particularly ^219^Rn, which is gaseous. By using porous zeolite nanoparticles as delivery system, between 90 and 95% retention of decay products was achieved (164). Furthermore, the nanoparticles were functionalized with a ligand that targets NK-1, a receptor overexpressed in glioma cells, providing selective cytotoxic effect *in vitro*. An alternative to loading the radionuclides in porous materials to minimize the release of daughters is encapsulating the α-emitters in shell structures. For instance, nanoparticle shells made of LaPO_4_ encapsulating ^223^Ra were able to retain up to 88% of the radionuclide (and its daughters) over 35 days (165).

**FIGURE 5:**
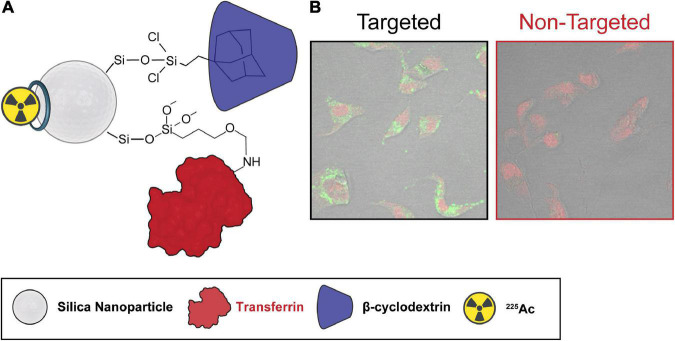
Silica nanoparticles as delivery vehicles for targeted alpha therapy. Silica nanoparticles were used as delivery vehicles of ^225^Ac for targeted alpha therapy. Transferrin and cyclodextrin were used as targeting and stabilizing agents, respectively. **(A)** Schematic representation, **(B)** cellular uptake with and without targeting agent. Adapted with permission from ref 139. Copyright 2020 American Chemical Society.

Similar strategies (i.e., encapsulation with porous nanomaterials or nanoparticle shells) have also been used to improve the retention of ^225^Ac daughters. For example, mesoporous silica nanoparticles, which have high surface-to-volume ratios because of their porous structure, allowing high radionuclide content per particle, were employed in ^225^Ac-based targeted alpha therapy against breast cancer cells (Figure 5) (166). The silica nanoparticle pores were loaded with ^225^Ac complexed by a hydroxypyridonate chelator, which improved radionuclide retention. The nanoparticle surface was functionalized with targeting agents, which promoted accumulation and cytotoxic effects in cancerous cells. Furthermore, *in vivo* studies in mice demonstrated that the nanoparticles enhanced radionuclide excretion, minimizing internal deposition. Alternatively, gold nanoshells made of ^225^Ac-doped La_0.5_Gd_0.5_PO_4_ cores and covered by thin gold shells displayed enhanced radionuclide retention (167). The gold nanoconstructs were further functionalized with antibodies and demonstrated targeting capabilities *in vivo*. A recent work by Karpov et al. further demonstrated the benefits of metal coating on radionuclide retention. The study reported that ^225^Ac encapsulated in silica cores and coated by gold or titania shells displayed no significant toxicity effects up to 10 days post-administration, as reveled by histological analysis (168). Furthermore, no radionuclide could be detected in non-targeted organs during that period of time. Beyond ^225^Ac, gold nanoparticles have also been used to deliver other α-emitters, such as ^211^At (169).

Lastly, titania nanoparticles radiolabeled with ^225^Ac and functionalized with peptide fragments targeting NK-1 receptors were used to selectively deliver the α-emitter to glioma cells (170). Alternatively, ^225^Ac was also incorporated into liposome-based nanoparticles, which could cross the blood-brain barrier and deliver the cytotoxic dose to glioblastoma cells through integrin α_*V*_β_3_-targeting (171, 172).

## 5 Challenges and future opportunities

Targeted alpha therapy has demonstrated to be an effective therapeutic strategy against a wide range of cancers, including tumors that were resistant to conventional treatments (26). Nevertheless, targeted alpha therapy has not become a gold standard treatment in oncology, as other therapies have, because of several challenges.

First, implementation of targeted alpha therapy in the clinic requires α-emitters being easily available and at reasonable costs, two conditions that are not currently met (173). For example, the annual production of ^225^Ac is around 75 GBq, which can only support a few hundred patients per year (174). No single production source is expected to individually achieve the sufficient scale for widespread use of ^225^Ac in the near future. Nevertheless, medium-energy proton irradiations of ^232^Th targets and high flux reactor irradiation of ^226^Ra and ^227^Ac targets are leading the efforts to yield enough ^225^Ac production for large patient population treatment (174). Overall, there are ongoing technical efforts (both in the private and public sectors) to overcome the production and supply issues associated with targeted alpha therapy radioisotopes (175). Those include developing new production avenues for these key medical radioisotopes (176–178).

Second, despite the absence of resistance mechanisms to α-radiation, cancerous cells may develop coping strategies against the delivery of the radionuclides, such as down-regulating the expression of surface proteins that are being targeted by the radiopharmaceuticals. Hence, expanding the current library of targeting agents and epitopes will be necessary to face tumors with acquired resistance, as well as cancers that inherently show low antigen expression.

Third, dosimetry calculations for the medical radioisotopes and daughters are still challenging because of the wide-range of factors that need to be considered. For example, the intra- and inter-tumor heterogeneity, including variation in antigen expression and vascularization, strongly affects the interaction between the radiopharmaceutical and the cancer cells. Hence, imaging techniques that provide this type of information are necessary, as well as modeling methods capable to account for all the different factors. Autoradiograph can image the tumor with high resolution, but it has to be done *ex vivo* (179). Some groups exploited the Ɣ-emission during ^225^Ac decay to image the radiopharmaceutical in the tumor by SPECT (180–182). Nevertheless, the low activities used during targeted alpha therapy render the imaging of ^225^Ac by SPECT very challenging (66). In this situation, theranostic pairs, where one radioisotope provides therapeutic benefits and the other one imaging/diagnostic information, are a valuable alternative. However, theranostic imaging does not provide information regarding the redistribution and impact of the daughters.

Four, the α-emitters, particularly those with multiple α-decays in their decay chain, are limited by the recoil effect, which causes the release of part of the daughters from the radiopharmaceutical (29, 183). This is problematic for two main reasons. First, the instability of the radiopharmaceuticals complicates the exact determination of their radiochemical purity. Second, the uncontrolled circulation and deposition of the daughters may damage healthy tissue and induce radiological poisoning. Because this issue is unlikely to be solved by new chelators, a novel method to minimize the release of the daughters is encapsulating the radionuclides in nanoconstructs (41). These nanoformulations, however, have been primarily tested in pre-clinical settings, and they are still far from being used in patients.

Regarding nanoformulations, it is also worth noting that nanoparticles are routinely used in the clinic for cancer therapy and imaging (e.g., Doxil, Abraxane, Hensify, Oncaspar, ^99m^Tc colloids) (184). In therapy, nanomedicines with long circulation times are usually preferred, as those allow for larger accumulation of the nanoformulations at the pathological sites, which tend to yield better therapeutic outcomes (185). In the case of targeted alpha therapy is unclear whether short or long circulation times should be favored, as longer circulation times could result in higher therapeutic effects but larger possibility of radionuclide internal deposition. Nevertheless, there are currently nanoformulation designs with long (e.g., Doxil, Hensify) and short (e.g., Cornell dots) circulation times either clinically approved or in clinical trials. Therefore, both pharmacokinetic designs are achievable, and future studies will have to define which option is better suited for targeted alpha therapy.

## 6 Summary and outlook

Targeted alpha therapy is a very promising treatment in oncology, since it can focalize highly cytotoxic doses to cancer cells, while sparring the surrounding tissue. This therapy relies on the delivery of α-emitters to the tumor sites guided by targeting agents. In this context, α-emissions are advantageous over other types of radiation, since they have higher linear energy transfers and shorter path ranges, yielding higher therapeutic performance with lower potential side effects. Despite the positive results of targeted alpha therapy in both pre-clinical and clinical studies, where tumors resistant to conventional treatments were partially or completely eradicated, there are still challenges that need to be addressed (e.g., radionuclide scarcity, precise dosimetry calculations, and daughter release from the drug) before targeted alpha therapy can be routinely used in patients.

In this comprehensive review, we have summarized the state-of-the-art in the development of radiopharmaceuticals for targeted alpha therapy, identifying their key structural components. The pros and cons of the different radionuclides (e.g., isotopes with one or multiple α-emissions in their decay chain) as well the targeting vectors (e.g., small molecules, peptides, and antibodies) commonly used in the field have been critically discussed. Moreover, we have highlighted ongoing strategies to overcome some of the main pitfalls the therapy currently presents, such as encapsulating the radionuclides in nanoformulations to prevent the release of the daughters. Lastly, we have discussed potential opportunities the field holds, which may contribute to targeted alpha therapy becoming a gold standard treatment in oncology in the future. Hence, we believe this review will assist other scientists to understand the current status of the field, allowing them to recognize promising research directions that may be important in the future.

## Author contributions

RP and RA wrote the manuscript. Both authors contributed to the article and approved the submitted version.
